# A systematic analysis of mitochondrial aminoacyl tRNA synthetase variants in a rare disease cohort

**DOI:** 10.1038/s41431-025-01990-y

**Published:** 2025-12-27

**Authors:** Thiloka E. Ratnaike, M. Eren Kule, Ida Paramonov, Leslie Matalonga, Kiran Polavarapu, Catarina Olimpio, Rita Horváth

**Affiliations:** 1 Department of Paediatrics, University of Cambridge, Cambridge, UK; 2 Department of Paediatric Neurology, University Hospitals Bristol and Weston NHS Foundation Trust, Bristol, UK; 3 Department of Physiology, Pharmacology and Neuroscience, University of Bristol, Bristol, UK; 4 Department of Clinical Neurosciences, School of Clinical Medicine, University of Cambridge, Cambridge, UK; 5 Koç University, School of Medicine, Istanbul, Turkey; 6 Centro Nacional de Análisis Genómico (CNAG), Barcelona, Spain; 7 Children’s Hospital of Eastern Ontario Research Institute, Ottawa, ON Canada; 8 Division of Neurology, Department of Medicine, The Ottawa Hospital, Ottawa, ON Canada

**Keywords:** Genomics, Neuromuscular disease

## Abstract

Mitochondrial aminoacyl-tRNA synthetases (mt-aaRSs) are a group of proteins encoded by nuclear DNA that play a crucial role in mitochondrial protein synthesis. Mitochondrial diseases caused by mt-aaRS variants are phenotypically heterogenous but often present with significant neurological features such as childhood-onset encephalopathy and seizures. As such, these conditions are a diagnostic challenge. We present an approach that systematically quantifies phenotypic similarity of individuals with an mt-aaRS variant to published cases, to aid variant interpretation, in RD-Connect—a large Europe—wide rare disease cohort. Across 98 individuals with a mt-aaRS gene of interest, we prioritised 38 individuals with 63 variants following bioinformatic and manual analyses. We additionally reviewed Exomiser prioritisation using a pre-defined gene list for neurological disorders within the RD-Connect Genome-Phenome Analysis Platform (GPAP). We were able to generate likely diagnoses in 11 individuals and VUS findings in 13 individuals, following careful phenotype similarity analysis using a phenotype-genotype dataset generated from 234 published individuals. Four of these 24 individuals did not have an Exomiser-ranked gene variant in the GPAP. Therefore, this approach, using individual-level curated phenotype-genotype data to support variant interpretation, can highlight potentially significant variants that may not be captured by current pipelines. This workflow can be replicated in other heterogeneous rare diseases to support clinical practice.

## Introduction

Mitochondria are essential for many cellular metabolic processes and are a vital source of energy. Variants in mitochondrial DNA (mtDNA) or in >400 nuclear-encoded mitochondrial proteins result in a group of rare disorders collectively known as mitochondrial diseases. These are genetic disorders that affect 1 in 4300 people worldwide [1–4]. One group of the nuclear-encoded mitochondrial proteins is mitochondrial aminoacyl tRNA synthetases (mt-aaRSs). Mt-aaRSs are enzymes that are essential for mitochondrial protein translation as they catalyse the charging of the mitochondrially transcribed tRNA with its cognate amino acid [5]. In mitochondria, there are 19 mt-aaRS genes responsible for the charging of each tRNA with the cognate amino acid (aminoacyl-tRNA), of these, 17 are specific to mitochondria, while 2 of them (*KARS*, MIM *601421 and *GARS*, MIM *600287) are bifunctional in both the cytosol and mitochondrial matrix. Pathogenic variants in mt-aaRS genes can lead to impaired protein translation, defective mitochondrial protein synthesis and subsequent combined respiratory chain dysfunction with cellular compromise, resulting in mitochondrial disease [6].

Mt-aaRS diseases are an important cause of paediatric mitochondrial disease, with approximately 300 reported cases worldwide. The recent increase in incidence of these conditions is most likely due to the increased availability and frequent use of exome or genome sequencing techniques [4, 7–9]. Pathogenic variants in each of the 17 mitochondria-specific mt-aaRS genes have been associated with disease and have distinct phenotypic presentations, with significantly variable age of onset and progression of disease. Most mt-aaRS deficiencies target specific areas of the central nervous system, such as the thalamus, brainstem, spinal cord and cerebellum, therefore resulting in a range of neurological symptoms including ataxia, seizures, hearing loss and spasticity [4, 7, 8, 10, 11]. Some mt-aaRS defects are accompanied by other organ involvement such as myopathy and lactic acidosis with anaemia (MLASA2, MIM #613561) commonly associated with *YARS2* (MIM *610957) variants [12], sensorineural hearing loss with ovarian failure (Perrault Syndrome, MIM #614926), caused by *HARS2* deficiency (MIM *600783) [13] or more complex syndromes such as HUPRA (Hyperuricemia, Pulmonary hypertension, Renal failure in infancy and Alkalosis, MIM #613845) [14] caused by *SARS2* (MIM *612804) [15]. In addition to the distinct phenotypes caused by each mt-aaRS-related disease, different variants in the same mt-aaRS gene can present with diverse phenotypes. An example for this is cardiomyopathy and encephalopathy observed with some individuals with the *AARS2 NM_020745.4:c.1774C*>*T p.(Arg592Trp)* variant [16, 17] and leukoencephalopathy with ovarian failure (MIM #615889) seen with other *AARS2* (MIM *612035) pathogenic variants [18].

Several mt-aaRS deficiencies are lethal in the first few months of life due to leukodystrophy or leukoencephalopathy; however, disease patterns can vary even in small cohort studies [7]. Early, accurate genetic diagnosis of these conditions is vital for tailoring management and expectations. The diagnosis for mt-aaRS-related disease is based on genetic testing, most frequently done by exome or genome analysis. Infants with mt-aaRS deficiencies frequently need acute care and, due to the lack of specific therapies, mainly receive symptomatic treatment [19]. While there is some evidence supporting beneficial effects of amino acid supplementation on cytosolic aaRS-related disease, studies on similar regimens for mt-aaRS deficiencies are much more limited [19, 20]. As the symptoms vary and the incidence is rare, the expert skillset in recognising mt-aaRS-related disease is uncommon. Limited access to genetic testing in underprivileged countries makes the diagnosis difficult and delays treatment. Therefore, it is important to create a systematic approach to diagnosing these conditions using next-generation sequencing technologies alongside structured phenotype data.

RD-Connect is a multidisciplinary project funded by the European Commission (FP7 grant), which enables the structured reanalysis of individuals with rare diseases, using the genome-phenome analysis platform (GPAP) [21]. The GPAP contains genomic datasets alongside phenotypic data in the form of human phenotype ontology (HPO) terms [22], which have been submitted by clinicians. These datasets have accelerated clinical bioinformatics approaches, within rare disease research projects, aimed at improving diagnostic yield and facilitating novel gene discovery [21].

In this paper, we aimed to identify mt-aaRS pathogenic variants associated with mt-aaRS-related disease within the RD-Connect GPAP. We present an approach to tackle the phenotypic heterogeneity in this rare disease cohort, where we have identified 38 individuals with 63 mt-aaRS variants across 10 mt-aaRS genes. Careful manual annotation, generation of an mt-aaRS-specific phenotypic dataset and incorporating phenotype analysis in the pipeline resulted in improved classification of variants and 11 likely new diagnoses.

## Subjects and methods

### Identification of individuals

Data on 10,935 individuals were visible to all registered and authorised users on the RD-Connect GPAP platform in July 2023. We filtered for affected unsolved individuals with homozygous mt-aaRS variants, gnomAD frequency <0.01, gnomAD homozygous allele count = 0, GPAP internal frequency<0.02 and high or moderate variant effect predictor (VEP) score. Additionally, we selected for individuals with two or more heterozygous mt-aaRS variants with gnomAD allele frequency <0.01, GPAP internal allele frequency <0.02 and high or moderate VEP score that do not have Benign or Likely Benign (B/LB) annotations in ClinVar. Individuals without phenotype data, single heterozygous variants or artefacts of sequence alignment were excluded from the dataset. We also excluded variants in genes where the proteins were not specific to mitochondria and affect cytosolic protein synthesis (e.g. *KARS* and *GARS)*.

### Variant curation

The candidate mt-aaRS variants identified in undiagnosed individuals from the RD-Connect GPAP database were individually classified according to the American College of Medical Genetics (ACMG) standards and guidelines for interpretation of variants [23] and Association for Clinical Genomic Science Best Practice Guidelines for Variant Classification in Rare Disease 2024 [24]. We compared our manual classification with the classification tools available in the Varsome [25] and Franklin [26] online platforms. Using the RD-Connect GPAP, Exomiser [27] was applied to each participant sample where a mt-aaRS variant was identified using a gene list compiled by the European Reference Network for Rare Neurological Diseases of 1,837 genes called ‘ERN-RND’, which is already available within GPAP. The rank of the mt-aaRS gene variants was documented to understand whether prioritised gene variants contained likely pathogenic (LP) or pathogenic (P) variants using our curations.

### Phenotype analyses—literature review

A literature search using PubMed (updated to December 11, 2023) was conducted to manually populate a reference database with published patient-centric data, using a previous approach [28], for individuals with variants in mt-aaRS genes. In brief, all clinically relevant text was manually mapped to HPO terms per published affected individual and their gene variants were also recorded after classifying these as LP or P according to ACMG criteria. In this study, we focused on the 10 mt-aaRS genes where LP or P variants were identified within the RD-Connect GPAP cohort.

### Phenotype similarity analysis

We conducted phenotype analyses between the reference database and the downloaded HPO list from hpo.jax.org (accessed January 2025) for the same mt-aaRS genes, using the ontologyX suite of R packages [29]. Shared HPO terms were assessed using HPO term lists from the reference database- where the HPO term appeared 5 or more times, and the downloaded HPO list- where the HPO term appeared 2 or more times, to ensure that well-reported terms were included.

The analysis of the patient data within the GPAP was conducted using the reference database. Initially, mean phenotype similarity scores were calculated using Lin’s similarity methodology, within the *ontologySimilarity* R package [29], for individuals within the mt-aaRS reference database and a heterogeneous cohort of diagnosed individuals within the GPAP. The diagnosed cohort of individuals included those with mtDNA diseases, nuclear-mitochondrial genes and other nuclear gene variants associated with wide-ranging neurological and other phenotypes. The mean phenotype similarity score for each individual was calculated from the probands with the same gene in the reference database. For individuals without a matching gene diagnosis, the phenotype similarity was calculated using the five most similar probands within the reference database to measure the highest possible phenotypic similarity between the individual and our reference dataset.

### Predictive value of the phenotype similarity score

To evaluate the predictive value of the mean phenotype similarity score in diagnosing mt-aaRS-related diseases, we developed two models: a Generalized Linear Model (GLM), which used logistic regression and a Random Forest (RF) classifier. The analysis was performed in R using the *caret* [30] and *pROC* [31] packages. The reference mt-aaRS dataset and the diagnosed cohort dataset were combined and individuals with mt-aaRS diagnoses were labelled as the positive class ‘mt-aaRS’, while all other diagnoses were designated as the negative class ‘Other’. The ‘Other’ class was randomly subsampled to achieve a balanced dataset of mt-aaRS: Other labels. The dataset was split into 80% training and 20% test sets.

For the GLM, a logistic regression model was trained to distinguish between ‘mt-aaRS’ and ‘Other’ diagnoses using the mean phenotype similarity score as the predictor variable. The training dataset was evaluated using 5-fold cross-validation implemented in the caret R package. The RF classifier was developed using mean phenotype similarity score, HPO count and average information content (IC) to assess their combined predictive value. The average IC was calculated across the HPO terms per individual using the *ontologySimilarity* R package [29]. The same training and test datasets were used as for the GLM. The RF model was built with 500 trees and 5-fold cross-validation was performed on the training dataset. Variable importance was evaluated using the *varImp* function from *caret* [30]. The optimal phenotype similarity threshold for predicting mt-aaRS diagnoses was identified using the Youden index [32] using both models and applied to the test set. The models were evaluated using accuracy, sensitivity, specificity and Area Under the Curve (AUC) as key metrics. Both models were compared using receiver operating characteristic (ROC) curves and AUC values with 95% confidence intervals were calculated using the DeLong method (*pROC* package) [31]. These were visualised using *ggplot2* [33].

### Investigation of undiagnosed individuals with mt-aaRS variants in GPAP

To prioritise individuals for further investigation, we revised the ACMG classification criteria by including the phenotype similarity scores that met threshold features as supported by the model evaluations. We manually reviewed the available phenotype data and individuals who were identified to have a high likelihood of mt-aaRS-related disease, based on the variant classification and structured phenotype assessment, were highlighted to their recruiting clinicians through the GPAP. Here, we report the likely diagnostic rate.

## Results

### Overall GPAP cohort evaluation for mt-aaRS variants

The initial queries were performed among the 10,935 individuals visible to all registered users on RD-Connect GPAP. The first analysis filtered for potential compound heterozygous variants with gnomAD frequency <0.01, with filters as described. This search yielded 145 variants in 117 individuals, across 16 genes.

We screened the 145 variants to identify which genes had variants that were possibly pathogenic, using ACMG criteria. One individual had variants in *IARS2* (MIM *612801) and *YARS2*, another in *AARS2* and *VARS2* (MIM *612802). The rest of the cohort had variants only in one gene. Following this screen, we excluded B/LB and false positive variants in the following genes: *FARS2* (MIM *611592) (*n* = 2), *HARS2* (*n* = 4), *PARS2* (MIM *612036) (*n* = 4), *SARS2* (*n* = 12), *TARS2* (MIM *612805) (*n* = 4) and *YARS2* (*n* = 8). Seven variants were deemed false positives due to mapping errors and poor-quality control parameters; 54 of 145 distinct variants were classified as B/LB by at least one of the tools, Varsome and Franklin. The initial ACMG classification identified LP/P variants in at least one of the variants (presumed compound heterozygous) in the following genes: *AARS2*, *CARS2* (MIM *612800), *DARS2* (MIM *610956), *EARS2* (MIM *612799), *IARS2*, *LARS2* (MIM *604544), *NARS2* (MIM *612803), *RARS2* (MIM *611524), *VARS2*, *WARS2* (MIM *604733). Therefore, these 10 genes were included in our study.

We identified 111 distinct variants presumed compound heterozygous in 98 patients in the 10 mt-aaRS genes in our cohort: *AARS2 (n* = *15)*, *CARS2 (n* = *8)*, *DARS2 (n* = *3)*, *EARS2 (n* = *18), IARS2 (n* = *13)*, *LARS2 (n* = *6)*, *NARS2 (n* = *8)*, *RARS2 (n* = *10)*, *VARS2 (n* = *24)*, *WARS2 (n* = *6)*. Variants that were considered artefacts due to the sequence alignment being too close (*n* = 13) were excluded without further analysis. Two variants in *AARS2*, initially presumed to be in a compound heterozygous state (*AARS2 NM_020745.4:c.1649G*>*C p.(Gly550Ala)*; and *NM_020745.4:c.1621G*>*A p.(Glu541Lys)*) were identified in 36 individuals in our cohort. However, following Integrative Genomics Viewer (IGV) analysis showing these in cis in the 36 individuals, these variants were excluded. One patient with another variant in *AARS2* found in combination with these variants was excluded. Additionally, another participant with two presumed compound heterozygous variants was excluded because there were no HPO terms available for them in GPAP, precluding further analysis. Overall, 18/111 variants were excluded.

**Fig. 1:**
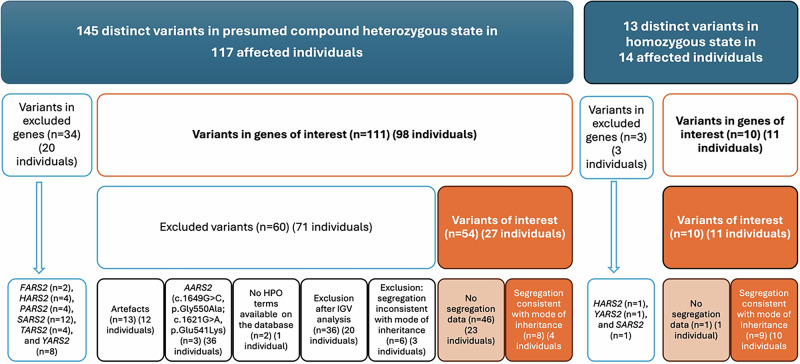
Filtering approach for mt-aaRS variants. Initial query within the RD-Connect Genome-Phenome Analysis Platform yielded 145 distinct variants found likely in a compound heterozygous position and 13 homozygous variants. Variants were excluded from the more uncommonly detected genes or where phenotype data were not available. After performing manual quality checks among the variants in genes of interest, there remained 54 distinct variants in the likely compound heterozygous cohort (27 individuals) and 10 homozygous (11 individuals) variants, a total number of 63 variants (38 individuals). HPO: Human Phenotype Ontology.

Further review of the remaining 93 distinct variants presumed compound heterozygous, in 50 patients using IGV analysis, quality control parameters and mapping errors, resulted in the exclusion of 36 variants in 20 individuals. *DARS2 NM_018122.5*:*c.142G*>*T p.(Val48Phe)* was found in two patients, but only one individual was included due to quality control metrics. The segregation analysis of the remaining 58 distinct variants presumed compound heterozygous, resulted in the exclusion of six variants in three individuals. One of the two individuals with the same variants (*IARS2 NM_018060.4:c.1488A*>*T p.(Leu496Phe)*, *NM_018060.4:c.2739T*>*G p.(Phe913Leu)*) was excluded due to lack of segregation, while the others remained in the final cohort with no segregation information available. Therefore, 54 variants were included in our final candidate list for possible mt-aaRS-related diseases in 27 individuals (Fig. 1).

A second analysis, filtering for homozygous variants with gnomAD frequency <0.01, gnomAD homozygous allele count equal to 0, with an internal GPAP frequency <0.02 and with high or moderate impact, identified 14 individuals with 15 homozygous variants. One individual had homozygous variants in both *DARS2* and *VARS2*. After excluding variants not within the 10 mt-aaRS genes of interest, there were 10 variants in 11 individuals remaining as the homozygous candidates for mt-aaRS-related conditions (Fig. 1). Overall, after bioinformatic and manual filtering approaches, 38 individuals were evaluated with 63 distinct variants in the mt-aaRS genes of interest.

### Variant curation

**Fig. 2:**
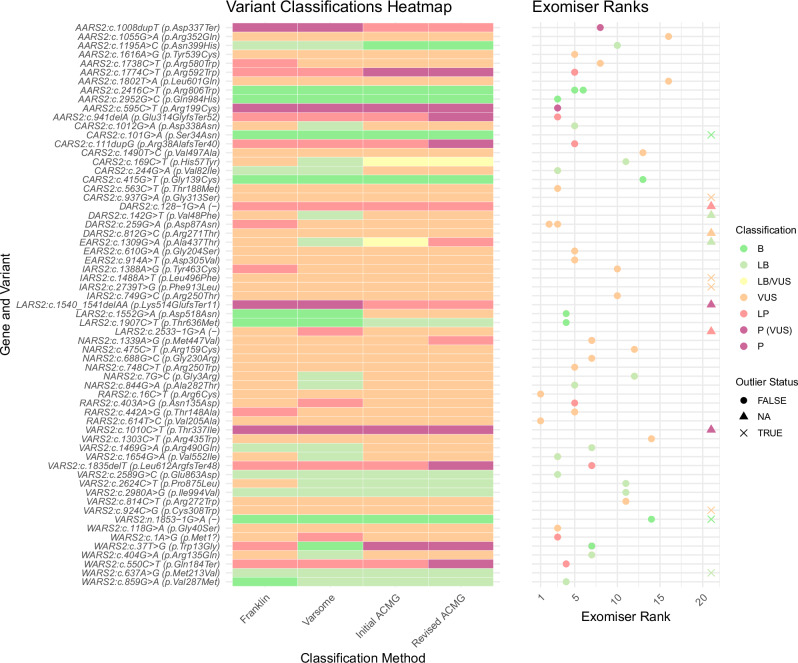
Comparison of mt-aaRS gene variant curation results using different methods for 38 individuals with 63 variants in genes of interest in RD-Connect GPAP. The ‘Initial ACMG’ category was the initial manual classification using American College of Medical Genetics (ACMG) criteria. Franklin and Varsome were used to compare results. The Revised ACMG includes the PP4 criteria where relevant, according to the use of phenotype similarity scoring, where a score ≥0.4 was PP4 (moderate) and ≥0.3 was PP4 (supporting). The scatterplot indicates the Exomiser results according to where the mt-aaRS gene-of-interest ranked using the ERN-RND gene list comprising 1837 genes. Genes ranked >20 in the list were classed as outliers; ‘x’ denotes where the case or variant was not ranked by Exomiser using the ERN-RND gene list; the remaining points are coloured by the overall classification. There was no obvious correlation between the LP or P variants and the Exomiser rank. B: Benign; ERN-RND: European Reference Network for Rare Neurological Diseases; GPAP: Genome-Phenome Analysis Platform; LB: Likely benign; LB/VUS: Likely benign/ Variant of uncertain significance; LP: Likely pathogenic; P: Pathogenic. ‘P (VUS)’ denotes variants which are LB/VUS when using ACMG criteria, but these are widely reported as pathogenic (i.e. hypomorphic allele).

The variant curation (Fig. 1) revealed that 11/38 participants carried LP or P variants in one of the 10 mt-aaRS genes. These were either homozygous or presumed compound heterozygous but not confirmed to be in trans (Fig. 2). To further ascertain pathogenicity, phenotypic similarity of the individuals was investigated by establishing a reference phenotype-genotype database for the 10 mt-aaRS genes.

### Phenotype analyses—literature review

Following a PubMed literature review, clinical data on 234 published individuals, from 87 published articles, were manually curated as HPO terms associated with autosomal recessive disease caused by variants in the 10 mt-aaRS genes of interest (Table 1).

**Table 1:** Summary of data collated for 10 mt-aaRS genes using PubMed literature review updated to 11/12/2023.

Gene	HGNC ID	Published individuals	Publications	Compound heterozygous or homozygous variants
*AARS2*	HGNC:21022	44	21	32
*CARS2*	HGNC:25695	6	5	4
*DARS2*	HGNC:25538	35	10	16
*EARS2*	HGNC:29419	28	12	23
*IARS2*	HGNC:29685	13	5	11
*LARS2*	HGNC:17095	19	6	14
*NARS2*	HGNC:26274	25	12	14
*RARS2*	HGNC:21406	25	11	18
*VARS2*	HGNC:21642	18	5	8
*WARS2*	HGNC:12651	21	8	16

The corresponding HGNC identifiers per gene, the number of individuals with phenotype-genotype data available, the number of publications and the number of distinct variant pairs (alleles 1 and 2) are recorded.

The HPO downloaded dataset (downloaded 26/01/2025) from hpo.jax.org was used to evaluate the known HPO-gene associations for the 10 mt-aaRS genes of interest. There were 336 non-redundant HPO terms associated with the 10 genes in the downloaded dataset and 957 HPO terms seen in the manually curated reference dataset, which is reflected by the differences in the comparison of common HPO terms between the two datasets (Supplementary Fig. 1).

**Fig. 3:**
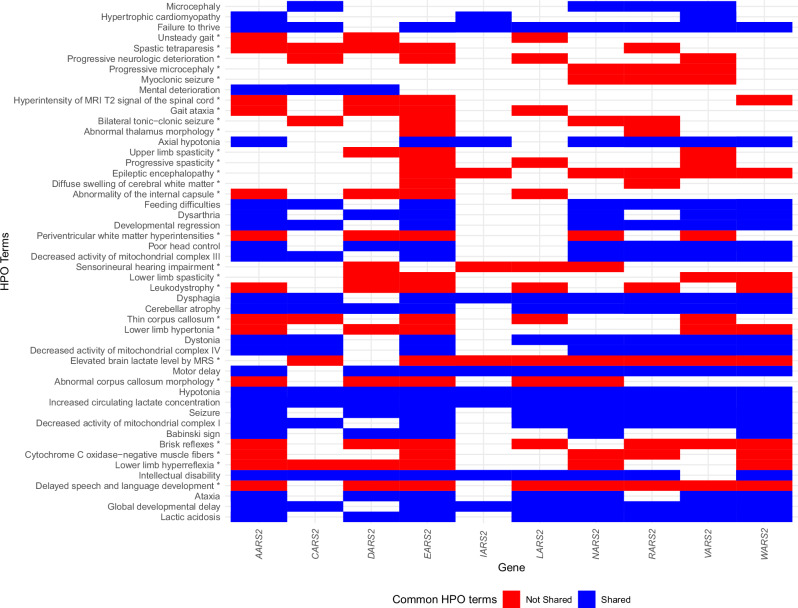
Shared terms between the downloaded HPO dataset and manually curated reference dataset for 10 mt-aaRS genes. The HPO Terms represent the terms seen >10 times per gene across both datasets. The asterisk shows HPO terms that are found commonly in the curated reference dataset (seen at least five times across the whole dataset) but not seen at least two times in the downloaded dataset. Red tiles indicate terms that are not shared and blue indicates shared terms between the two datasets. HPO: Human Phenotype Ontology.

The shared terms between the datasets were evaluated (Fig. 3). There were six terms found in the downloaded dataset and not in the reference dataset: ‘Long philtrum’, ‘Abnormal speech pattern’, ‘Cleft palate’, ‘Flexion contracture’, ‘Neurodegeneration’, ‘Nonimmune hydrops fetalis’. However, on further investigation, variations of all terms, apart from ‘Long philtrum’, appeared in the reference database. There were more HPO terms seen five or more times in the reference database and not seen in the downloaded dataset (coded as ‘Not shared’ in Fig. 3), which suggested that the reference database contained more discriminative terms, which would aid phenotype similarity mapping in this study.

### Phenotype similarity analysis

**Fig. 4:**
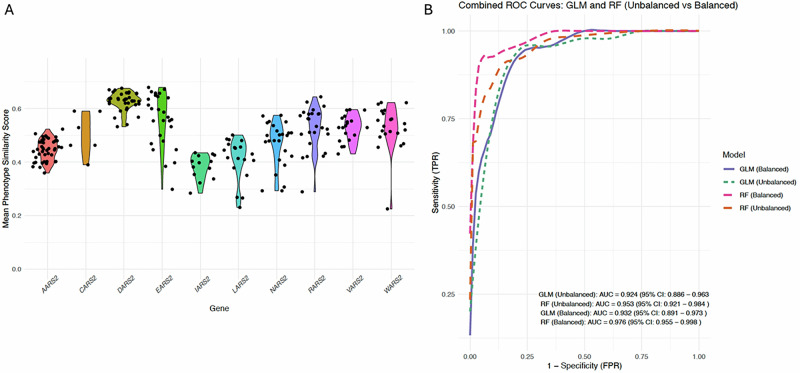
Mean phenotype similarity evaluations in the reference and other datasets. **A** The mean phenotype similarity scores per person, by gene, across the mt-aaRS reference dataset. **B** Receiver operating characteristic (ROC) plot using the training and test datasets for modelling the mean phenotype similarity score to detect individuals with mt-aaRS-related diseases. Area under the curve (AUC) values and the corresponding confidence intervals (CI) are displayed on the graph for the balanced and unbalanced datasets evaluated through the generalised linear (GLM) and random forest (RF) models.

The mean phenotype similarity scores were assessed as previously described [28] and Fig. 4 shows that the scores were consistently >0.2 for published cases across the 10 mt-aaRS genes and only 9 published individuals had a mean phenotype similarity score <0.3. Individuals with a *DARS2* diagnosis tended to cluster closely together with higher mean phenotype similarity scores, suggesting less heterogeneous phenotypes in this group with predominant neurological features. This contrasted with individuals with *IARS2* variant diagnoses (Fig. 4A) and is likely due to the different clinical presentations, including sideroblastic anaemia, dysmorphic features, MRI and EEG abnormalities, within the group of 13 published individuals with *IARS2* (Table 1).

### Predictive value of the phenotype similarity score

A total of 1520 molecularly diagnosed individuals from the GPAP were included in this study, consisting of 64 with mtDNA diseases, 118 with nuclear-mitochondrial gene diagnoses (including three with mt-aaRS deficiencies) and 1338 with other nuclear gene diagnoses. This dataset was combined with the reference mt-aaRS dataset (*n* = 234), resulting in a cohort of 1754 individuals. The data were partitioned into a training set (*n* = 1406; 191 mt-aaRS and 1215 ‘other’ diagnoses) and a test set (*n* = 348; 46 mt-aaRS and 302 ‘other’ diagnoses). This partitioning preserved the relative rarity of mt-aaRS-related diseases in the testing set while allowing model training with a larger and balanced representation of diagnoses in the training set.

The GLM, when applied to the test set, achieved an accuracy of 85.5% (95% CI: 79.72–91.34%), with a sensitivity of 93.0% and specificity of 81.4%. The positive predictive value (PPV) was 76.7% and the negative predictive value (NPV) was 95.1%. The balanced accuracy was 85.5% and the Kappa value was 0.693, indicating moderate agreement between predictions and true diagnoses (Supplementary Table 1). The GLM demonstrated strong discriminatory power with an AUC of 0.932 (95% CI: 0.891–0.973) in the balanced dataset. The RF model demonstrated superior performance, achieving an accuracy of 94.2% (95% CI: 90.31–97.34%) on the test set. It showed a sensitivity of 90.7% and specificity of 96.5%. The PPV was 88.6%, while the NPV was 95.9%. The balanced accuracy was 91.3% and the Kappa value was 0.811, indicating strong agreement between predicted and actual diagnoses (Supplementary Table 1). The RF model exhibited high discriminatory power, with an AUC of 0.976 (95% CI: 0.955–0.998) in the balanced dataset (Fig. 4B). The mean similarity score was the most influential predictor in distinguishing ‘mt-aaRS’ from ‘other’ cases, with a variable importance score of 112.9, the inclusion of HPO count and average IC did refine the model’s ability to differentiate positive cases, but they had lower contributions with variable importance scores of 41.2 and 36.4 respectively.

When comparing unbalanced data, the GLM achieved an AUC of 0.924 (95% CI: 0.886–0.963), while the RF model achieved an AUC of 0.953 (95% CI: 0.921–0.984), as seen in Fig. 4B. Both models effectively identified mt-aaRS-related diseases; however, the RF model consistently outperformed the GLM in balanced accuracy and specificity across datasets. Both models, using unbalanced and balanced datasets, showed that the optimal mean phenotype similarity score was 0.363–0.365, using Youden’s index to identify the best threshold value (Table 2). Therefore, for the evaluation of the undiagnosed RD-Connect GPAP dataset, we used a mean phenotype similarity score of ≥0.3 to suggest supporting phenotype similarity and ≥0.4 to suggest moderate phenotype similarity.

**Table 2:** Optimal thresholds and corresponding evaluations for different models.

Model	Threshold	Mean similarity score	Sensitivity	Specificity
GLM (Unbalanced)	0.152	0.363	0.935	0.821
RF (Unbalanced)	0.143	0.363	0.913	0.871
GLM (Balanced)	0.409	0.365	0.930	0.814
RF (Balanced)	0.699	0.365	0.907	0.965

Optimal thresholds, corresponding mean similarity scores and the resulting sensitivity and specificity values for the Generalised Linear Model (GLM) and Random Forest (RF) model under both balanced and unbalanced conditions.

### Investigation of undiagnosed individuals with mt-aaRS variants in GPAP

We explored the use of phenotype similarity scores alongside variant annotation to evaluate undiagnosed individuals within RD-Connect GPAP. In the cohort of 38 undiagnosed individuals who carried rare recessive mt-aaRS gene variants, the following phenotype data were available: the minimum HPO term count was 1 (*n* = 12) and maximum was 13 HPO terms (*n* = 1), the mean number of HPO terms per individual was 4.08, with a median of 3. The 38 individuals were evaluated using the revised variant pathogenicity classifications, which incorporated the phenotype similarity evaluations. There were 9 individuals with initial ACMG criteria meeting LP or P criteria in at least one allele, with the other variant classified as VUS or higher. With the addition of the PP4 criteria (PP4 supporting for mean phenotype similarity score ≥0.3 < 0.4 and PP4 moderate for mean phenotype similarity scores ≥0.4), 11 individuals met criteria for further investigation based on variant pathogenicity. Furthermore, there were initially 14 individuals with a VUS in presumed biallelic or homozygous states and with the updated PP4 criteria, the revised variant pathogenicity classifications showed one less individual with presumed biallelic VUS. Note that one individual had a homozygous *DARS2 NM_018122.5:c.812G*>*C p.(Arg271Thr)* variant, which was classified as a VUS and a homozygous *VARS2 NM_020442.6:c.1010C*>*T p.(Thr337Ile)* variant, which was classified as pathogenic, so overall there were 24 individuals with gene variants for possible or definite further investigation.

The gene variants and HPO data were ranked by Exomiser using the ERN-RND 1837 genes list within GPAP. Of the 24 individuals with suspected mt-aaRSs genetic diagnoses or further investigation required, 17 had variants that ranked within the top 10 gene variants. We found that there were 4/24 (17%) individuals without an Exomiser-ranked gene variant (Fig. 1, Supplementary Table) within the GPAP, which highlights that our careful evaluation of the phenotype and genotype data associations provides added benefit. Overall, the addition of the individual-level phenotype similarity score upgraded the classification of seven variants to LP or P (Fig. 1) in six individuals, with an overall yield of 11/98 individuals (11.2%) with likely diagnoses.

## Discussion

Phenotypic spectrum using individual data from publications can be very helpful in determining pathogenicity of variants; however, often this data is interpreted manually, on a case-by-case basis. Here, we built a workflow to assess phenotype similarity scores using individual-level published data to improve the diagnostic yield of next-generation sequencing in a large genomic database, utilising information from participants across Europe. This leveraged the phenotype similarity between published individuals with a diagnosis of one of the 10 most common mt-aaRS-related diseases and undiagnosed individuals with variants in these 10 mt-aaRS genes in our study. Recent work using a similar approach also successfully identified previously undiagnosed individuals with mtDNA disease diagnoses within a large Europe-wide cohort [34]. There was phenotypic variability within the reference dataset, which is representative of the phenotypic heterogeneity published in mt-aaRS-related diseases. Applying GLM and RF models to a heterogeneous dataset of individuals with molecular diagnoses, including mitochondrial diseases caused by mtDNA and nuclear genes, showed that mean phenotype similarity scores were highly discriminative of mt-aaRS-related diseases. We manually evaluated each of the samples within the RD-Connect GPAP using ACMG criteria between two independent curators, to ensure robust validation of the initial filtering approach and then applied the phenotype similarity threshold as PP4 Supportive (phenotype similarity score ≥0.3 and <0.4) or PP4 Moderate (phenotype similarity score ≥0.4) to generate the revised ACMG classification.

We confirmed 11/38 (28.9%) affected individuals with biallelic LP or pathogenic variants explaining their phenotype. There were a further 13 variants classified as VUS, with a mean HPO count of 4, suggesting limited data in this group. In these cases, we would recommend additional family history and segregation of phenotype with genotype; however, parental samples or additional family samples were unfortunately not available for this study. Examples of the clinical utility of the similarity score implementation included the ability to upgrade variants to LP or P status. In one individual with homozygous *EARS2 NM_001083614.2*:*c.1309G*>*A p.(Ala437Thr)*, PP4 Moderate was added due to the high phenotype similarity with other published individuals with *EARS2-*related disorders. This resulted in the variant being upgraded to LP and our recommendation to further investigate this individual and their family. Another individual, with *NARS2 NM_024678.6:c.1339A*>*G p.(Met447Val), NM_024678.6:c.688G*>*C p.(Gly230Arg)* variants had high phenotype similarity, which meant we were able to upgrade *NARS2 NM_024678.6:c.1339A*>*G p.(Met447Val)* to LP, but the *NM_024678.6:c.688G*>*C p.(Gly230Arg)* variant remained classified as VUS based on ACMG criteria. The *DARS2 NM_018122.5:c.259G*>*A p.(Asp87Asn)* homozygous variant was found in two individuals with high phenotype similarity, but current evidence suggests PM2 Moderate and BP4 Supporting according to ACMG criteria and therefore, the contribution of this *DARS2* variant to the phenotype remains uncertain, even when adding PP4 Moderate. Additional functional data or larger cohort segregation studies could potentially upgrade this variant to LP.

A limitation of our study is the use of just 10 mt-aaRS genes to create the reference database. This was due to time constraints, but also due to the focus on genes where LP or P variants had been identified during the initial filtering process, therefore creating a clinically impactful database to aid variant annotation in these genes. We excluded variants in *KARS* (*n* = 5) and *GARS* (*n* = 5), as they act in both cytoplasm and mitochondrial translation, hence named bifunctional aminoacyl tRNA synthetases. They also differ from the other mt-aaRS as they can cause disease in both an autosomal dominant and an autosomal recessive manner [35]. Further work is required to add the remaining mt-aaRS genes to our reference database. Automated natural language processing tools to gather individual-level and variant-level HPO data from published text could enrich reference databases like ours. Current work is ongoing to develop and refine large language models that can make this process more efficient [36]. However, there are issues with aligning text to HPO terms [36], which have implications for rare disease reference datasets that need to be accurate to infer genotype-phenotype associations.

A well-known limitation in rare disease research is the scarcity of published cases with diagnosed genetic variants and subsequently the lack of curated databases to enable streamlining of the diagnostic process for the individual and their family. However, we noticed that the quality of the HPO data within RD-Connect and the difficulty in feeding back to the participants or their carers directly made it more difficult to resolve the diagnosis. More streamlined communications with the recruiting clinician in rare disease studies such as this may further improve the diagnostic rates and help resolve VUS.

We have developed a useful phenotype database for 10 of the mt-aaRS-related diseases, where variants in these genes were detected in GPAP. This work, done in collaboration with clinicians, geneticists and bioinformaticians, has led to upgrading seven variants to LP or P, which has practical consequences for the patients. It was difficult to resolve the remaining VUS without further information from the family or functional data. Detecting a VUS cannot confirm a genetic diagnosis and would not allow prenatal or preimplantation genetic testing. By providing further evidence for the pathogenicity of VUS and reclassifying them as LP in mt-aaRS disease enables families to access further reproductive options, including prenatal diagnosis and pre-implantation genetic testing, should they wish to. Endeavours to evaluate the pathogenicity of variants will continue to require close partnership between bioinformaticians and clinicians, to ensure accurate diagnoses are provided. Our data collection was aligned to the data collection processes of MitoPhen, which is a database that contains manually curated genotype-phenotype data on individuals with mtDNA diseases [28]. Recent work has shown that examining the MitoPhen database can be used to answer various clinical or scientific questions on phenotypes and treatment effects [37]. Further work would include completing the mt-aaRS genotype-phenotype reference database for the remaining seven rare mt-aaRS genes (*FARS2, MARS2, YARS2, SARS2, HARS2, TARS2, PARS2*). Further research will be needed to understand the refined processes required to distinguish the pathogenicity of variants with high phenotype similarity but still classified as VUS, for example, with RNA sequencing or family segregation studies.

## Supplementary information

Supplementary Information

## Data Availability

Access to pseudonymized phenotypic information for all individuals and their genetic variants is possible through RD-Connect GPAP (https://platform.rd-connect.eu/), on completion of registration and approval by the independent RD-Connect Data Access Committee. The mt-aaRS reference dataset will be available as part of the latest MitoPhen (version 2) data release [38].
