# Supplementary material for: A systematic analysis of mitochondrial aminoacyl tRNA synthetase variants in a rare disease cohort
Source: Eur J Hum Genet. 2025 Dec 27;34(3):395–403. doi: 10.1038/s41431-025-01990-y (PMC12963482; doi:10.1038/s41431-025-01990-y)
Supplement: Supplementary file 1 — Supplementary Information [file 41431_2025_1990_MOESM1_ESM.docx]

**Supplementary Information**

**Results**

**Supplementary Figure 1 Common HPO terms seen in downloaded and manually curated datasets.** A: HPO terms seen at least 3x in the downloaded HPO dataset. B: HPO terms seen at least 15x in the manually curated ‘reference’ dataset. The HPO terms are ordered by frequency within the entire dataset with the least frequent terms at the top of the tile plots, and the terms are coloured by root system, using ontologyIndex R package.

| **Model** | **Balanced Accuracy** | **Sensitivity** | **Specificity** | **Kappa** | **Sample Size** |
| --- | --- | --- | --- | --- | --- |
| **GLM (Unbalanced)** | 0.713 | 0.457 | 0.970 | 0.501 | 348 |
| **RF (Unbalanced)** | 0.810 | 0.630 | 0.990 | 0.712 | 348 |
| **GLM (Balanced)** | 0.855 | 0.837 | 0.872 | 0.693 | 129 |
| **RF (Balanced)** | 0.913 | 0.907 | 0.919 | 0.811 | 129 |

**Supplementary Table 1: Model performance for identifying mt-aaRS-related diagnoses across unbalanced and balanced datasets**. Balanced accuracy, sensitivity, and specificity are reported for each model: Generalized Linear Model (GLM) and Random Forest (RF). Kappa indicates agreement between predicted and actual classifications, where higher values signify stronger agreement. Results are shown for unbalanced datasets (n = 348) and balanced datasets (n = 129). The Random Forest model trained on the balanced dataset demonstrated the highest balanced accuracy (0.913), sensitivity (0.907), and specificity (0.919), suggesting superior overall performance compared to other models.
